# Supplementary material for: Whole Proteome Clustering of 2,307 Proteobacterial Genomes Reveals Conserved Proteins and Significant Annotation Issues
Source: Front Microbiol. 2019 Feb 28;10:383. doi: 10.3389/fmicb.2019.00383 (PMC6403173; doi:10.3389/fmicb.2019.00383)
Supplement: Supplementary file 1 [file Image_1.pdf]

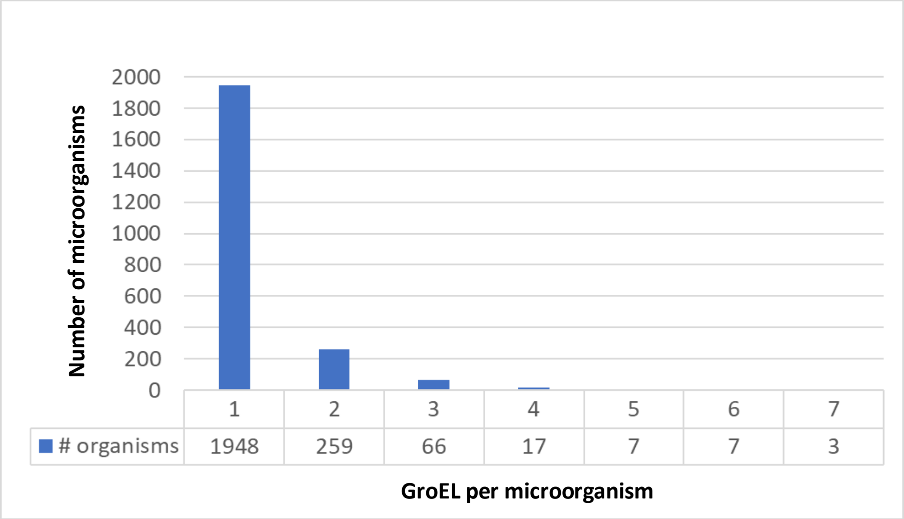

Supplementary Figure 1. Number of *groEL* sequences per microorganism. This graph shows the number of organisms in the study that had one or more *groEL* genes. All organisms had at least one gene, and it was rare for an organism to have more than two genes.
