# Supplementary material for: Whole Proteome Clustering of 2,307 Proteobacterial Genomes Reveals Conserved Proteins and Significant Annotation Issues
Source: Front Microbiol. 2019 Feb 28;10:383. doi: 10.3389/fmicb.2019.00383 (PMC6403173; doi:10.3389/fmicb.2019.00383)
Supplement: Supplementary file 5 [file Table_4.pdf]

Supplementary Table S4. PolA in Endosymbionts.

**Endosymbiont Species Name**	**Number with Full PolA**	**PolA.1**	**No PolA**	**Total**
Buchnera aphidicola	2	16	0	**18**
Candidatus Portiera aleyrodidarum	0	0	9	**9**
Candidatus Carsonella ruddii	0	0	8	**8**
Wolbachia	8	0	0	**8**
Blochmannia endosymbiont	5	1	0	**6**
Candidatus Hodgkinia cicadicola	0	0	4	**4**
Baumannia cicadellinicola	2	1	0	**3**
Candidatus Tremblaya	0	0	3	**3**
Candidatus Nasuia deltocephalinicola	0	0	2	**2**
Candidatus Profftella armatura	0	2	0	**2**
Moranella endobia	2	0	0	**2**
Wigglesworthia glossinidia	0	2	0	**2**
Arsenophonus symbiont of Lipoptena fortisetosa	1	0	0	**1**
Candidatus Evansia muelleri	0	0	1	**1**
Candidatus Ishikawaella capsulata Mpkobe	1	0	0	**1**
Candidatus Riesia pediculicola USDA	0	0	1	**1**
Candidatus Tachikawaea gelatinosa	0	1	0	**1**
Candidatus Zinderia insecticola CARI	0	0	1	**1**
Coxiella endosymbiont of Amblyomma americanum	1	0	0	**1**
Coxiella-like endosymbiont	1	0	0	**1**
endosymbiont of Acanthamoeba sp. UWC8	1	0	0	**1**
endosymbiont of Bathymodiolus septemdierum str. Myojin knoll	1	0	0	**1**
endosymbiont of unidentified scaly snail isolate Monju	1	0	0	**1**
secondary endosymbiont of Ctenarytaina eucalypti	1	0	0	**1**
secondary endosymbiont of Heteropsylla cubana	1	0	0	**1**
Serratia symbiotica str. 'Cinara cedri'	1	0	0	**1**
**Total**	**29**	**23**	**29**	**81**
