# Supplementary material for: Whole Proteome Clustering of 2,307 Proteobacterial Genomes Reveals Conserved Proteins and Significant Annotation Issues
Source: Front Microbiol. 2019 Feb 28;10:383. doi: 10.3389/fmicb.2019.00383 (PMC6403173; doi:10.3389/fmicb.2019.00383)
Supplement: Supplementary file 8 [file Data_Sheet_1.pdf]

Supplementary File

Supplementary File 1: Regular Expressions for Searching Clusters.

1) Command to search for GroEL:

- `egrep '[G|g][R|r][O|o][E|e][L|l]'`

2) Command to search for RpoB and RpoB':

- `egrep '[R|r][P|p][O|o][B|b]'`
- `egrep '[R|r][N|n][A|a] polymerase.*beta'`

3) Command to search for DNA polymerase I:

- `egrep '[D|d][N|n][A|a] [P|p]olymerase [A|a][lpha]*[^A-Za-z]'`
- `egrep '[D|d][N|n][A|a] [P|p]olymerase [I|i][^A-Za-z]'`
- `egrep '[P|p][O|o][L|l][ ]*[A|a|I|i][^A-Za-z]'`
